# Managing and querying gene expression data using Curray

**DOI:** 10.1186/1753-6561-5-S2-S10

**Published:** 2011-05-28

**Authors:** Hasan Jamil, Aminul Islam

**Affiliations:** 1 Department of Computer Science, Wayne State University, Michigan, USA

## Abstract

**Background:**

In principle, gene expression data can be viewed as providing just the three-valued expression profiles of target biological elements relative to an experiment at hand. Although complicated, gathering expression profiles does not pose much of a challenge from a query language standpoint. What is interesting is how these expression profiles are used to tease out information from the vast array of information repositories that ascribe meaning to the expression profiles. Since such annotations are inherently experiment specific functions, much the same way as queries in databases, developing a querying system for gene expression data appears to be pointless. Instead, developing tools and techniques to support individual assignment has been considered prudent in contemporary research.

**Results:**

We propose a gene expression data management and querying system that is able to support pre-expression, expression and post-expression level analysis and reduce impedance mismatch between analysis systems. To this end, we propose a new, platform-independent and general purpose query language called *Curray*, for Custom Microarray query language, to support online expression data analysis using distributed resources. It includes features to design expression analysis pipelines using language constructs at the conceptual level. The ability to include user defined functions as a first-class language feature facilitates unlimited analysis support and removes language limitations. We show that Curray’s declarative and extensible features nimbly allow flexible modeling and room for customization.

**Conclusions:**

The developments proposed in this article allow users to view their expression data from a conceptual standpoint - experiments, probes, expressions, mapping, etc. at multiple levels of representation and independent of the underlying chip technologies. It also allows transparent roll-up and drill-down along representation hierarchies from raw data to standards such as MIAME and MAGE-ML using linguistic constructs. Curray also allows seamless integration with distributed web resources through its LifeDB system of which it is a part.

## Background

Much of the attention in microarray data management so far has been in laboratory information management systems (LIMS) as opposed to comprehensive microarray data management systems (MADAMS). This is because the main focus so far has been to bring chip level data to a usable level so that expression profile can be generated to select the differentially expressed genes for onward analysis. Once differentially expressed genes are at hand, the lower level data does not have much usefulness and hence, does not demand much management consideration. Another reason is that the use of the differentially expressed genes from the gene expression data is highly context dependent and seems to be completely orthogonal to the management and maintenance of the expression data. So, researchers have viewed such analysis as distinct applications as opposed to data manipulation within the gene expression “database” in its traditional sense.

LIMS systems such as BASE [1], MIMAS [2], EzArray [3], and ArrayTrack [4] process raw expression data to bring it to the level where differential expression analysis can be performed. This step is usually called the pre-expression analysis. In the expression analysis level, popular tools such as Bioconductor/R [5], or GSEA [6] algorithm are used to find the differentially expressed genes. The most common post-expression analysis includes pathway analysis, functional annotation, comparison, validation and publication of obtained data. These operations are needed to ascertain the value of the gene list of interest, and to weed out genes that may appear in the list by mere chance.

The critical issue here is that each of the the post-expression analysis needs are widely subjective. There is no one simple or single method that can be used to perform such an analysis, e.g, functional annotation. The outcome of the analysis depends greatly upon what method is used. Researchers often discover errors in the data at this stage, or find it necessary to revise how differential expression is computed. Often there is a need to iteratively update or recalibrate probe level or chip level data in this process, and discover the right choice of the tools to compute the differentially expressed set of genes. Since the data needed are spread across multiple systems and platforms, the associated the context switch makes the process difficult. In this way, computing differentially expressed genes potentially is a truly investigative and iterative process. We argue that researchers need a single platform system that will allow them to manage such revisions and changes with a few key strokes.

### Need for a declarative language for expression data

The lack of declarative query languages for microarray data manipulation is remarkable, given that the community is keenly aware of its potential. The emergence of MIAME [7] actually acknowledges the fact that we need a high level view for expression data that hides the lower level physical view of the microarrays. ArrayExpress tool suit [8] provides library support for the migration of Affymterix, Agilent and Illumina data to its FGEM (Final Gene Expression Matrix) format, from which conversion to MAGE-ML [9] or MAGE-TAB is routine. While systems such as Bioconductor/R allows analysis support for Affymterix in the form of a rich collection of libraries, it also supports data in emerging formats such as MIAME, MAGE-ML, MAGE-TAB and ArrayExpress.

Although tool support for migration or data management at a higher level exists, researchers seldom use this level to process their data, except when they are forced to do so to deposit the data in public repositories such as ArrayExpress. One possible reason may be the perceived loss of control over the data due to format migration and abstraction (lower level representation obviously has more detailed information that a Biologist may need when a closer look at the data is needed). Regardless of the reasons why advantages offered by standards are not always exploited at the application level, the practice wastes substantial time and resources. Had there been a data management system that allowed the flexibility to move up and down the data abstraction hierarchies and offered the control a user seeks, they would have overwhelmingly preferred to use the standard representation.

Two new approaches toward expression data management and analysis can be envisioned. The first approach is obvious - development of a new data management system with a declarative query language for data manipulation. The second is to move into package based data analysis such as Bioconductor. We are not aware of any serious high profile attempt to develop a declarative language for microarray data management other than the early project Expresso at Virginia Tech [10]. Although this project attempted to create a declarative approach to managing and querying expression data, it did so for a LIMS system which is not at the level of Curray. Thus no significant advances have been made in terms of a declarative query language for data manipulation in Expresso. The ROOT system [11] on the other hand advocated an alternative to Bioconductor for massive data sets, including microarray expression data. The question now is whether it is possible to develop a data management system and a query language for expression data that does not depend upon specific tools such as Bioconductor, and allows flexibility in terms of data management. As a byproduct, it will also offer seamless integration with other declarative systems such as SQL, BioFlow [12,13] and Prolog, for example, to allow access to conventional data and reasoning engines. We believe the answer is in the positive.

## A canonical model for microarray data

**Figure 1:**
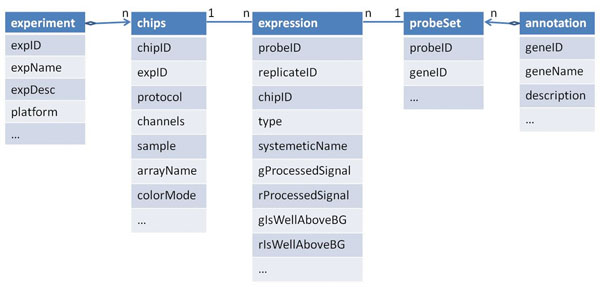
Conceptual relationship of objects in a microarry experiment.

Before we consider a model for microarray data, let us revisit how microarray chips are designed at the conceptual level. For simplicity, we will refer to only Agilent chips although the discussion uniformly applies to Affymetrix and Illumina chips, and the emerging Solexa technology. Usually, an experiment *e* is designed to gather expression values for different excitation conditions for a particular set of genes *G*. These genes may come from a set of control samples *G_c_* and any number of experimental sample sets *G_e_* for the same set of genes *G*. In other words, ∀*g*(*g* ∈ *G* ⇔ *g* ∈ *G_c_*), and ∀*g*, *e*(*g* ∈ *G* ⇔ *g* ∈ *G_e_*). Each gene *g* ∈ *G* is divided into *k_g_* number of segments, called probes *p*, denoted *p_g_i__*, such that . One experiment is usually composed of multiple chips (one per condition *c* ∈ *C*), and often an experiment is replicated several times (*n*) to ensure correctness. Therefore, the total number of chips *T* is equal to *n* × |*C*|. Each chip contains spots or expression values in the form of two colors (green and red) for several probes under one excitation condition. Each spot on a chip represents one probe of a gene under a given condition that the chip represents. However, probes are replicated *m* number of times within the chip again for ensuring error free expression reading, such that some form average of all *m* replicates of a probe *p* represents its true expression. Finally, the total number of spots on a chip is equal to *m* × ∑_*g*∈*G*_*k_g_* Conceptually, a microarray experiment is as simple as this and the diagram in Figure 1 can be used to adequately capture this relationship.

Although the underlying relationship of the chips, probes, genes and experiments is complex, our goal in modeling experiments is to allow a uniform and very high level view of a set of experiments in which researchers will neither have to think in terms of such a complex relationship, nor deal with the platform difference between the underlying technologies, i.e., Agilent, Illumina and Affymetrix. Once the conceptual model is understood, the technology specific details can be handled by the system through an appropriate mapping. Conceptually then, users should have the ability to refer to experiments as a single object of interest, as a set of genes in an experiment, or gene expression values without any reference to any specific technology. Users should also be able to select a subset of an experiment based on any condition that describes that experiment. For example, they should be able to ask questions such as *“what is the set of differentially expressed genes in experiment epilepsy for conditions X*, *Y and Z”*, or *“what is the set of differentially expressed genes in experiment epilepsy for conditions X*, *Y and Z*, *and without considering the red channel and probe replicates having expression higher than θ”*? These questions of course assume that we have a procedure to compute differential expression in some coherent manner, and so on.

We are now ready to present our model. Let ***I***_*e*_, ***I***_*g*_, ***I***_*p*_, ***I***_*r*_ and ***I***_*c*_ are sets of experiment, gene, probe, probe replicate and chip identifiers respectively, and ***A*** be a set of attributes with associated domain ***D***. Then, an experiment *e* ∈ *E* ⊆ ***I***_*e*_ × *D*_1_× … × *D_k_* such that *D_i_* are domains corresponding to attributes *A_i_****A***, and for ∀*e*_1_, *e*_2_ ∈ *E*, of the form <*e*_1_, *d*_1_,…,*d_k_* > and , . In other words, experiments are unique and identifiable using the experiment ID. In a similar way, we define a chip as an association with experiments as *C* ⊆ ***I***_*e*_ × ***I***_*c*_ × *D*_1_ × … × *D_l_* over the attributes *A_l_*. However, in the set *C*, ***I***_*c*_ is unique (no two tuples have the same chip ID). Similarly, we define a gene as a set of unique objects *G* ⊆ ***I***_*g*_ × *D*_1_ × … × *D_m_* such that *A_m_* is the set of attributes defining them, and ***I***_*g*_ is the set of unique identifiers. Since probes are segments of genes, we define probes as an association *P* ⊆ ***I***_*g*_ × ***I***_*p*_ × *D*_1_ × … x *D_n_*, where attributes *A_n_* describe the probes, and ***I***_*p*_ is the key. Finally, an expression is defined as a many-many association between probes and chips as *Expr* ⊆ ***I***_*p*_ × ***I***_*c*_ × ***I***_*r*_ × *D*_1_ × … × *D_j_*. Since we allow multiple replicates of a probe in the same chip, the identifying elements are ***I***_*p*_ × ***I***_*c*_ × ***I***_*r*_.

### Mapping heterogenous experiments to canonical model

**Figure 2:**
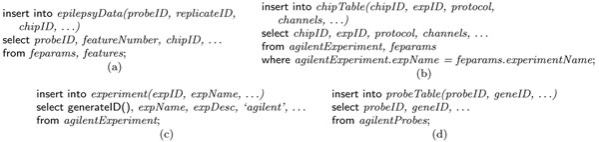
Mapping LIMS tables to Curray expression tables using SQL.

Once the canonical model is decided, we are left with designing a user transparent mapping for each of the microarray experiment types (Agilent, Affymetrix and Illumina). Since the model for each of the technologies is standard and the models come with well defined schemes, designing a mapping ***M*** : *F* → *M*, where *F* is the set of format representation possible, each defined as a set of tables, and *M* is a singleton containing our canonical model as defined. For the sake of brevity, in Figure 2 we partially show a possible mapping from Agilent format to Curray as a set of SQL expressions that can be cast into a function.

## Recipe for Curray

To view all expression data uniformly, we plan to make a clear separation between the conceptual components of all expression data and their platform (technology, class and type) related components so that at the application level, queries can be asked in uniform ways without referring to lower level details. To assign proper meaning to applications, we use rules to define concepts so that the meaning of a concept becomes context dependent and interpreted accordingly at run time. To offer control of data to the user, we also allow drill down and roll up type of concepts on expression data. For example, the Affymetrix library [14] available in R/Bioconductor can summarize the probe set intensities to form one expression value for each gene. Usually probe level data can be used for quality control, RNA degradation assessments, different probe level normalization and background correction procedures, and flexible functions that permit the user to convert probe level data to expression measures. However, this function is not available for MAGE-ML data. So if that information is of interest, queries need to be performed over Affymetrix level data. Providing support for one or the other can often be considered limiting, and may motivate users to stay close to lower level data that offers them the control they feel they may need.

We view Curray as a sub-language of our recently proposed language BioFlow [12] in our LifeDB database management system for Life Sciences applications [13]. Curray provides all necessary constructs needed to handle expression data in the context of biological science while BioFlow support basic data manipulation, data integration and representation mismatch (XML, relational, text, etc.). We divide representation and manipulation of expression data into three categories in Curray - data definition, concept definition and expression data manipulation language. We take advantage of the fact that almost all microarray data are read only, and hence, no updates are expected. Consequently, views created from base data are also read only. We now present the proposed language Curray using several example fragments.

### Data definition language

Curray data definition language allows defining an experiment at the raw data level as it is delivered from LIMS systems, called limstable, and at the conceptual level for end users, called expressiontable. The experiments at the conceptual level are considered to be “base” level or “expression” format. All other levels are viewed relative to this base level. Therefore, the LIMS level would be considered a drill-down, and MAGE-ML level a roll-up from base level. Although in an earlier section we have outlined a canonical representation of expression objects and tables, Curray leaves open what tables can be considered a limstable or an expressiontable in terms of their structures. This is done to accommodate arbitrary tables into the framework in which there may be a need for using existing tables as expressions. Consequently, Curray does not reserve specific scheme, or identifiers for key fields. But it does insist on the table structure as shown in Figure 1. The following example clarifies this further.

**Figure 3:**
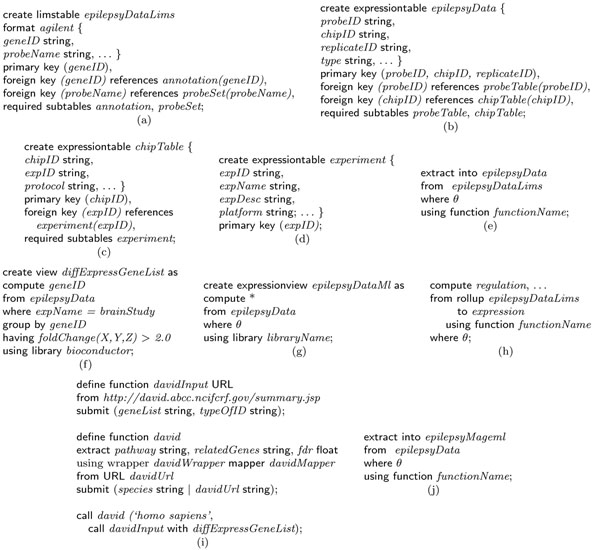
Data definition and data manipulation statements in Curray.

Assume that we have one raw gene expression data set from a LIMS system, called *epilepsyDataLims*, generated from brain tissue samples in Agilent format. However, the expression information of the samples is spread over three tables, including two other tables *annotation* and *probeSet*. The create limstable *epilepsyDataLims* statement in Figure 3(a) creates a composite table of these three tables in which table *epilepsyDataLims* is the root table, and tables *annotation* and *probeSet* are children of the root table, expressed through the required subtables *annotation*, *probeSet* clause. This composite object view of tables holds true for Curray tables created using limstable or expressiontable options, although these are traditional SQL tables and can be used in SQL statements. Notice that these definitions induce a dependency graph and join path among the set of tables that constitute the *epilepsyDataLims*. In this case, the composite view allows access to the sub tables as a large table formed using a natural join of all three tables transparently to the users, and thus, users are not required to think in terms of so many tables and their interrelationships.

This lower level object view of experiments is captured at the conceptual level by the create expressiontable *epilepsyData* in Figure 3(b) in a way that mimics the structure in Figure 1. In this case, the root table is *epilepsyData*, and the tables are of depth three as the sub-tables have their own sub-tables. Once an expression table or LIMS table has been defined, it can be populated in two principal ways. First, if a data set requires conversion from an external format such as text or XML to LIMS table format or base format, we use extract statement as shown in Figure 3(e). We use extract to also up-convert data from a lower view to an upper view, i.e., from *agilent* format to base format. The where clause allow filtering of unwanted objects in LIMS level data in *epilepsyDataLims*. The declaration format *agilent* in Figure 3(a) makes it possible to appropriately choose a map function to transform the objects in the LIMS table to Curray table *epilepsyData*, again as a composite object. The using function clause in Figure 3(e) is optional. It allows specialized functions, and in its absence, the system selects the default function for the mapping (possibly the one shown in Figure 2). Since the format for *Agilent* (or *Affymetrix*) data is standard, it needs to be defined and the mapping functions developed only once in the database. Once defined, we can statically create a MAGE-ML version of the *Agilent* table *epilepsyData* as shown in statement 3(j) in a similar way. It is important to note here that extract is a format conversion statement. It can also convert data from Curray formats (LIMS table and base format) to external formats such as MAGE-ML in XML. Again, similar to text data, to process data in MAGE-ML format, we will need to convert it to one of the Curray formats.

Note that there are numerous functions for converting *Affymetrix* and *Agilent* format to MAGE-ML format and we could choose one based on system preference. For the purpose of quality assurance, the output of *functionName* must match format definition in the format clause. Since we are not aware of any function that can create a lower level view from a higher level view, for example from MAGE-ML to *Affymetrix*, we postulate that it is not that useful to drill down dynamically. But it is certainly possible to imagine a dynamic conversion to a higher level view such as *epilepsyData* in base format from *Agilent* at query time as shown in statement 3(h) in which we are using a qualifier rollup in front of a table name to convert it to base format. The only difference is that this conversion will not be materialized. Since dynamic drill down is not feasible in the absence of suitable map functions, the user must query the lower level representation that is always part of the database, such as the *epilepsyDataLims* table, when needed. However, if and when reverse map functions become available, an equivalent drill-down option can be specified as drill-down *epilepsyDataMl* to *agilent* using *functionName*. If dynamic drill down becomes possible, we can then define drill down to any format. As a consequence, we can achieve platform transparency by being able to move from one format to the other. The definition suit above actually makes it possible to view the collection of tables that define *epilepsyData* as a single unit connected internally as a graph. These connections using join path can also be established by BioFlow’s link and combine type object aggregation and record linkage constructs.

### Data manipulation language

Data manipulation in Curray is accomplished using its native statements for querying basic expression data and using BioFlow for complex data processing involving local or remote data repositories and tools. A more detailed discussion on how BioFlow can be used may be found in [12,15]. The basic expression functions in Curray statements follow the suggestions in [16] that advocates four basic functionalities – class discovery, class comparison, class prediction and mechanistic studies. In general, Curray supports expression and post-expression level analysis through compute statement, as shown in Figure 3(f), and with the aid of create expressionview statement, as shown in Figure 3(g). compute functions in a way similar to SQL’s select statement, the only difference being that it accepts a composite expression table and possibly returns an expression table (to the extent the compute list retains the expression table structure). If insufficient number of attributes is retained in the compute list, it may degenerate into a relational table as in Figure 3(f). It is possible to show that the four types of functions Quackenbush [16] insists that a data management system for microarrays should perform, can be framed using Curray and BioFlow fully declaratively.

The statement 3(f) states that a fold change analysis needs to be performed and all genes that have a fold change greater than 2.0 should be returned in a brain study experiment using the *foldChange* function in *bioconductor* library. The using library clause in compute statements allow selecting analysis functions available in popular packages. In the current version of Curray, we have included Bioconductor/R as a library and users are able to choose all the functions this package supports. These functions are available in both the where and having clauses. The second statement in 3(i) actually uses the online database DAVID for a pathway analysis where false discovery rate (FDR) is used as cutoff value for the gene list in *diffExpressGeneList* computed using 3(f), where we show the BioFlow define function statement that implements the DAVID pathway function. It may be noted here that all these statements follow SQL’s compositional completeness property and hence can be composed to an arbitrary level of nesting. A similar statement can be developed for GO term analysis using DAVID without much effort.

A final note about the Curray statements is that although Curray has specialized constructs not native in SQL, the tables it creates are all traditional SQL tables. So, they can be used in any SQL statements as appropriate. The only difference is that when these tables (limstable or expressiontable) are used in traditional SQL sentences, their Curray specific interpretations are not applicable and standard interpretations apply. Also, before we conclude this discussion, we must mention that any complex analysis that is not covered by the basic Curray statements can be easily performed using BioFlow. The most enabling feature in Curray is in the way expression data is interpreted in the create expressiontable and create expressionview statements, and how they are organized behind the scene so that users need not worry about the details. Furthermore, the SQL-like feeling supported in Curray is interesting in which analysis functions are blended in through the using library option in the compute statements. In summary, Curray supports data definition and view definition through create limstable, create expressiontable, create expressionview, and extract into statements. The data manipulation is supported using only one new statement called compute that works similarly to the traditional select statement.

## Comparison with R/Bioconductor

The argument for designing a declarative language such as Curray for microarray data hinges upon the fact that query languages such as SQL are more palatable to end users than scripting languages such as Perl, BioPerl or R. In such declarative querying environments, it is possible to hide significant amount of details, and users focus on what to find, and not on how to find it. We will demonstrate the usefulness of Curray over R, in particular, using an example on epilepsy research in our lab.

**Figure 4:**
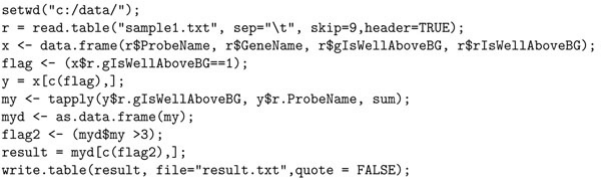
R script for present/absent analysis using green channel.

One of our epilepsy research required the development of two channel Agilent custom microarrays from brain samples of patients with epilepsy, and from normal people without epilepsy as control. Like many other chip design projects, it was discovered during analysis that some of the chips were not in good condition due to faulty hybridization, and as such, a separate set had to be designed and fabricated in another facility. Thus in this experiment, it was necessary to ascertain the goodness of the arrays by running a presence/absence (of probes) analysis. The expectation was that the control probes should be present in all chips as should all probes of a control gene. However, all probes of test genes were not expected to be present in all chips, because if they did, it would mean that those probes did not convey any information. Hence, presence/absence analysis classified probes as good or bad, and could be useful for future design of custom arrays that will allow discarding bad probes, thus making arrays more useful. Due to the modified chip status, adjustments were made as follows. Since red channel in the control data set (normal brain samples) was corrupted, it was dropped from any analysis. A gene was considered present if the majority of the probes were present either in single or bi-color. Under this altered condition and difference in analysis criteria, a custom R script such as the one in Figure 4 would be needed.

**Figure 5:**
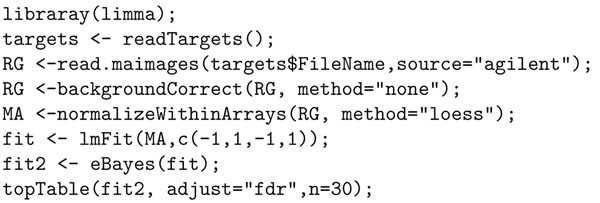
R script for computing differentially expressed genes in Agilent platform.

**Figure 6:**
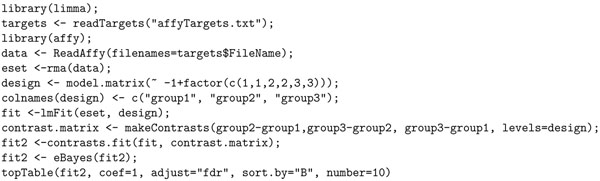
R script for computing differentially expressed genes in Affymetrix platform.

Our contention is that it requires significant skills and knowledge of R to develop such scripts. Not only that, doing simple tasks such as computing differential gene expression of an experiment requires significant expertise. For example, to compute the differentially expressed genes in R for our Agilent experiments, one will need to write the script in Figure 5, and the script in Figure 6, if the experiment is in Affymetrix. None of these scripts are as simple and intuitive as the query in Figure 3(f), written in Curray. In Curray, the user need not be aware that the experiment was in Agilent, Affymetrix or Illumina, for example.

## Examples of complete gene expression studies using Curray

We discuss a complete gene expression study using Curray so that the readers can have an intuitive feeling about the usefulness and the capabilities of the language. The first example is about checking the quality of the probes in an experiment; and the second is about computing the fold change of genes. Since gene expression analyses often require other data and tool applications, most real expression studies in Curray will require more than Curray features alone. In the examples to follow, we show how Curray statements, in conjunction with BioFlow and SQL statements, help declaratively conceptualize an analysis involving gene expression. It should be understood that in the context of gene expression and many Life Sciences analyses, a query usually means a set of steps that involves multiple queries in the traditional sense.

### Goodness of the probes

The basic idea of the analysis is to determine the quality of probes in an experiment. Probes in microarrays can be divided into two broad categories – control and test. To calculate the quality of probes, we can proceed as follows.

**Figure 7:**
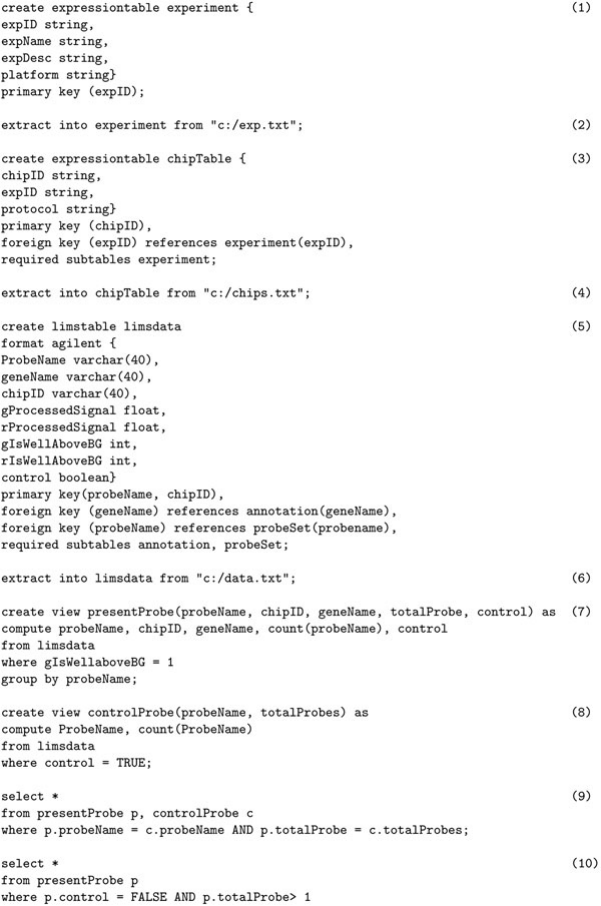
Curray script for computing the good probes.

A control probe is of good quality if all replicates of that probe in a chip are present. However, for a test probe, it must be present in at least one replicate in order to be considered good. The set of Curray expressions in Figure 7 extracts the set of acceptable probes. The view in statement (7) counts the number of times test probes are present in a chip, and the view in statement (8) counts the number of replicates of control probes . These views are used to compute the good control probes and test probes in statements (9) and (10) respectively.

### Fold enrichment analysis

**Figure 8:**
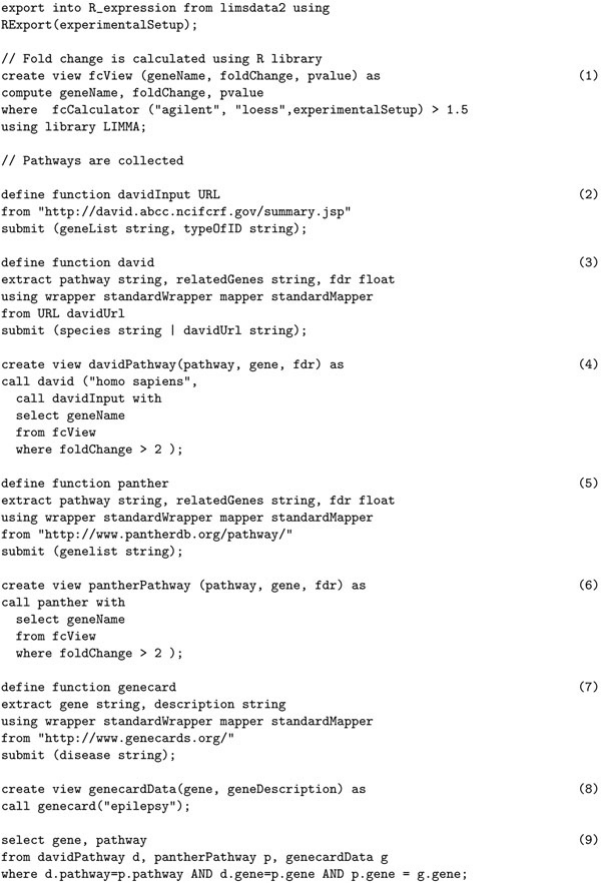
Fold Enrichment Analysis in Curray.

Computing fold enrichment is one of the most critical steps in many microarray experiments. By calculating fold change, we can cluster genes based on their expression and relative expression between experimental conditions. The whole experiment may be divided into three main parts: (i) compute fold change and isolate top *n* genes for next step, (ii) carry out a pathway enrichment analysis, and finally (iii) do a go term enrichment of the top *n* genes. In the discussion to follow with reference to Figure 8, we are assuming that proper extract, create limstable and create expressiontable statements have been included as appropriate.

Statement (1) in Figure 8 computes the fold change and collects the genes that cross a threshold. Statement (4) calls the functions defined in statements (2) through (3) to interact with DAVID web site which takes as input the list of genes that has passed threshold and collects enriched pathways and pathway related genes. A similar analysis is completed in statements (5) and (6) using the Panther database. Statement (8) calls the function defined in statement (7) which collects all genes related to particular disease which is “epilepsy” in the example. Statement (9) computes the final list of genes that are enriched both in Panther Pathway database and David Database (KEGG pathways) and have correlations to target disease in literature.

## A translational approach to implementation

**Figure 9:**
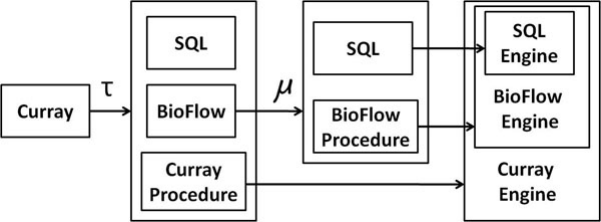
Translation procedure for Curray scripts to BioFlow statements.

The current implementation of Curray is based on a translational approach. Figure 9 shows the functional relationships between the various components of the system at a high and abstract level. Since Curray contains BioFlow specific statements, we use the transformation function *τ* to translate Curray statements to either SQL statements, BioFlow statements, or to Curray specific procedures that neither BioFlow or an underlying SQL engine would recognize – i.e., some implementation of the extract statement. Similarly, at the BioFlow engine level, many BioFlow statements cannot be implemented directly in SQL, and hence, we require a BioFlow processing engine that is capable of complementing SQL toward implementing BioFlow. Once Curray statements are mapped to either Curray procedures, SQL or BioFlow sentences, we transmit all BioFlow statements to BioFlow engine for another level of translation to SQL (or BioFlow procedures). The grammar for our complete set of Curray statements and the translation algorithms (for create expressiontable, create limstable, create expressionview compute and extract) statements may be found in Additional File 1. However, we do not discuss the translation procedures for BioFlow statements to SQL statements in this paper. Readers may consult BioFlow related articles in [12,13,15,17] for more the details.

## Results and discussion

Curray has been implemented as a stand alone expression data management system front-end for SQL using the MySQL database management system. Since Curray is part of BioFlow, it has a significant advantage over contemporary expression data management systems including Bioconductor/R because BioFlow and Curry are both SQL like text based query languages, built as front ends for MySQL, and BioFlow is capable of supporting workflow design and data integration over distributed network resources. Since Curray is a prototype and believed to be one of its kind, we did not concern ourselves too much with its execution efficiency. This is because regardless of the fact that a translational approach adds slight inefficiency to the overall implementation, it is still significantly better than the alternative – piecewise manual computation. When a graphical interface for Curray is implemented, and users are able to develop applications using this interface, the slight inefficiency due to translations will become inconspicuous.

## Conclusions

We have demonstrated that declarative querying of gene expression data at any level is possible and doing so yields significant benefits in terms of time and ease of application of development. Most importantly, most gene expression, post-expression analyses require integration with online analysis tools and databases in a computational pipeline. Most contemporary systems support such features very little, if at all. Curray, on the other hand can support all in one single platform. The developments proposed in this article allow users to view their expression data from a conceptual standpoint – experiments, probes, expressions, mapping, etc. at multiple levels of representations and independent of the underlying chip technologies. Curray also allows transparent roll-up and drill-down along the representation hierarchies from raw data to standards such as MIAME and MAGE-ML using linguistic constructs.

## Author contributions

Hasan Jamil designed the data model and language features of Curray while Aminul Islam designed the Curray system architecture and implemented the system.

## Competing interests

The authors declare no conflict of interests.

## Supplementary Material

Additional file 1 Curray grammar and translation algorithms Click here for file
