# Supplementary material for: Managing and querying gene expression data using Curray
Source: BMC Proc. 2011 May 28;5(Suppl 2):S10. doi: 10.1186/1753-6561-5-S2-S10 (PMC3090758; doi:10.1186/1753-6561-5-S2-S10)
Supplement: Additional file 1 — Curray grammar and translation algorithms [file 1753-6561-5-S2-S10-S1.pdf]

Curray grammar

In this section we present the CFG for all Curray statements. In the CFG below anything that begins with lower case letters are considered as non-terminals and anything all capitals are considered as terminals,

curray : (*statement*;)*

statement : (*createTable* |*compute* |*extract* |*expressionView*)

createTable : CREATE *tableType tableName* [FORMAT *format*]

lCurlyBrace parameterList rCurlyBrace

[primaryKey ,

(foreignKey ,)*

REQUIRED SUBTABLES (*tableName*)+]

tableType : EXPRESSIONTABLE |LIMSTABLE

format : AGILENT |AFFYMETRIX |MAGEML

primaryKey : PRIMARY KEY *lBrace identifier*+ *rBrace*

foreignKey : FOREIGN KEY *lBrace identifier*+ *rBrace*

REFERENCES *tableName lBrace identifier*+ *rBrace*

parameterList : *identifier dataType* [, *parameterList*]

compute : *computeSimple* |*computeRollup*

computeSimple : COMPUTE *attribute*+ |*all*

FROM *source*

WHERE *conditions*

[GROUP BY *attribute*+]

[HAVING *conditions*]

[USING (LIBRARY *libraryName* |FUNCTION *functionName*)]

computeRollup : COMPUTE *attribute*+

FROM ROLLUP *source* TO (*expressionTable* |*format*)

[USING (LIBRARY *libraryName* |FUNCTION *functionName*)

WHERE conditions]

conditions : *identifier operator values*

|*functionName lBrace attribute** *rBrace*

|*identifier operator conditions*

extract : EXTRACT INTO *tableName*

FROM *source*

WHERE *conditions*

USING FUNCTION *functionName*

expressionView : CREATE EXPRESSIONVIEW *viewName* AS *compute*

operator : $= | < | > | \leq | \geq$

all : *

identifier

tableName

viewName

source

attribute

functionName

libraryName

expressionTable : *literal*

lBrace : (

rBrace :)

lCurlyBrace : {

rCurlyBrace : }

values : *alpha-numeric-string*

dataType : *sql-data-types*

Translation algorithms

Algorithm 1 CreateTable

INPUT: *table*, *type*, *format*, *params*, *pKey*, *fKey*, *rTables*
OUTPUT: *sql* — An SQL statement

```
fParams := loadFormat(format)
if fParam ⊂ params then
  if rTables ≠ ∅ then
    invalid := validate(rTables)
    if invalid = TRUE then
      return Required Tables Not Initialized
    end if
  end if
  sql = create table table (
  for p ∈ params do
    sql += p
  end for
  sql += )
  if pKey ≠ “” then
    sql +=pKey
  end if
  if fKey ≠ “” then
    sql += fKey
  end if
  return sql
else
  return Format Mismatch
end if
```

Algorithm 2 Extract

INPUT: *table*, *condition*, *source*, *fName*, *fAttributes*
OUTPUT: *sql* — An SQL statement

```
exceuteSQL( call source destTable fAttributes)
attributes := getColumns( destTable)
sql := insert into table (
for p ∈ attributes do
   sql += p
end for
sql += )
sql += select
for p ∈ attributes do
   sql += p
end for
sql += from destTable
sql  += where condition
return  sql
```

Algorithm 3 ComputeSimple

INPUT: *outAttributes*, *source*, *wCondition*, *gAttributes*, *hCondition*, *fName*, *fAttributes*
OUTPUT: *sql* — An SQL statement

```
script := extractScript( fName)
script := updateScript( fAttributes)
destTable := runScript(script)
sql += select
for p ∈ outAttributes do
   sql += p
end for
sql += from destTable
sql += where wCondition
sql += having hCondition
sql += group by
for p ∈ gAttributes do
   sql += p
end for
return  sql
```

Algorithm 4 ComputeRollup

INPUT: *attributes*, *source*, *format*, *condition*, *fName*
OUTPUT: *sql* — An SQL statement

```
script := exteactScript( fName)
script := updateScript( source, format)
destTable := runScript(script)
sql += select
for p ∈ attributes do
    sql += p
end for
sql += from destTable
sql += where condition
return sql
```

Algorithm 5 ExpressionView

INPUT: *viewName*, *source*
OUTPUT: *sql* — An SQL statement

```
attributes := getColumns(source)
sql := create view viewName (
for p ∈ attributes do
    sql += p
end for
sql += ) as
sql += select
for p ∈ attributes do
    sql += p
end for
sql += from source
return sql
```
